# Extrachromosomal circular DNA drives dynamic genome plasticity: emerging roles in disease progression and clinical potential

**DOI:** 10.7150/thno.111765

**Published:** 2025-05-25

**Authors:** Bin Shi, Ping Yang, Huaijin Qiao, Daojiang Yu, Shuyu Zhang

**Affiliations:** 1 Laboratory of Radiation Medicine, West China School of Basic Medical Sciences & Forensic Medicine, Sichuan University, Chengdu 610041, China.; 2 Department of Laboratory Medicine, Affiliated Hospital of Zunyi Medical University, Zunyi 563006, China.; 3 The Second Affiliated Hospital of Chengdu Medical College, China National Nuclear Corporation 416 Hospital, Chengdu 610051, China.; 4 Medical College of Tibet University, Lasa 850000, China.

**Keywords:** extrachromosomal circular DNA (eccDNA), genomic plasticity, oncogene amplification, cancer progression, liquid biopsy biomarkers.

## Abstract

Extrachromosomal circular DNA (eccDNA) has emerged as a dynamic and versatile genomic element with key roles in physiological regulation and disease pathology. This review synthesizes current knowledge on eccDNA, covering its classification, biogenesis, detection methods, biological functions, and clinical implications. Once considered rare anomalies, eccDNAs are now recognized as major drivers of oncogene amplification, genomic plasticity, and therapeutic resistance, particularly in cancer. EccDNA subtypes such as microDNA, double minutes, and ecDNA are defined by their structural, genomic, and pathological features. EccDNAs originate through diverse mechanisms including DNA repair, chromothripsis, breakage fusion bridge cycles, and apoptosis, occurring in both normal and stressed cells. Advances in long-read and single-cell sequencing, CRISPR-based synthesis, and computational tools have improved detection and functional analysis. Functionally, eccDNAs contribute to transcriptional amplification, activate immune responses through cGAS-STING signaling, and facilitate intercellular communication. They are found across a range of tissues and disease states—including cancer, cardiovascular, neurological, autoimmune, and metabolic disorders—and serve as both biomarkers and regulatory elements. We introduce the concept of the stress selection theory, which proposes eccDNA as an adaptive reservoir that enhances cellular fitness in response to environmental and therapeutic pressures. Despite growing insights, challenges remain in understanding tissue-specific roles, achieving clinical translation, and standardizing methodologies. Emerging tools in multi-omics, spatial biology, and artificial intelligence are expected to drive future breakthroughs in precision medicine.

## Introduction

Deoxyribonucleic acid (DNA), the primary carrier of genetic information, exists in two distinct forms: linear and circular. In eukaryotic organisms, linear DNA is organized into chromosomes, with telomeres playing a crucial role in maintaining genomic stability during replication and segregation. Defects in telomere maintenance are often linked to genomic instability, a feature commonly associated with aging and cancer progression 1. In contrast, circular DNA provides structural stability and replicates independently of chromosomal machinery. This form is predominantly found in prokaryotes, where circular genomes and plasmids confer important adaptive advantages, such as antibiotic resistance 2. In eukaryotes, circular DNA is also present in organelles like mitochondria, where it plays a pivotal role in energy metabolism 3. In addition to these well-known forms, the discovery of extrachromosomal circular DNA (eccDNA) in eukaryotic cells has opened new avenues for understanding genomic plasticity, gene regulation, and disease mechanisms 4-5.

The first report of eccDNA appeared in 1965, when Bassel and Hotta observed circular DNA in boar sperm and wheat embryo cells using electron microscopy 6. At the time, eccDNA was regarded as a rare and biologically insignificant anomaly. However, it is now recognized as a widespread genetic element in a variety of eukaryotic species, including plants, animals, and humans 7. Recent advances in high-throughput sequencing technologies have revealed the pervasive presence of eccDNA in both normal and tumor tissues, implicating it in key processes such as oncogene amplification, immune modulation, and genomic instability 8-9. Notably, eccDNA has been identified as a driver of oncogene amplification in cancers, contributing to tumor heterogeneity, progression, and resistance to therapy 10-11.

The formation of eccDNA involves several distinct mechanisms, including errors in DNA repair, recombination events, and chromothripsis 1,12-14. Its formation is strongly influenced by cellular stress and genomic instability, with distinct patterns emerging under both physiological and pathological conditions 15-16. Despite significant progress in understanding its biology, several critical questions remain: What triggers the preferential formation of eccDNA in specific genomic regions? How does eccDNA interact with chromosomal DNA to influence cellular processes? What factors govern its stability and inheritance within cells? Resolving these questions is crucial for fully understanding the biological functions of eccDNA and its implications in various diseases.

In this review, we provide a comprehensive overview of current eccDNA knowledge, including its classification, mechanisms of formation, biological roles, and relationships with other molecular entities. We also explore the clinical potential of eccDNA as a diagnostic biomarker and a therapeutic target, with a particular emphasis on its role in cancer. Importantly, we propose the “stress selection theory,” originally formulated by our group, which posits that eccDNA formation is not merely a byproduct of genomic instability but rather a selective and adaptive process wherein cells preferentially retain or amplify certain eccDNAs that confer survival or proliferative advantages under stress conditions. This theory is supported by accumulating experimental evidence showing that DNA-damaging agents such as ionizing radiation, cisplatin, and doxorubicin significantly increase eccDNA abundance in cancer and other proliferative cell types, suggesting a stress-responsive mechanism of eccDNA generation and selection 17-20. Mechanistically, stress-induced eccDNA may originate from genomic regions enriched in oncogenes, repetitive elements, or enhancer hubs, and their retention may enable cells to escape cell cycle checkpoints, resist apoptosis, or rapidly reprogram gene expression 21,13. The stress selection theory offers a unifying model to explain why certain eccDNA species, especially those carrying oncogenes or stress-response genes, are enriched in tumors, senescent tissues, or inflamed environments. By synthesizing current knowledge and highlighting key areas of uncertainty, we aim to offer new insights into the biological and clinical significance of eccDNA.

## Discovery history of eccDNA: foundations and advances

**Figure 1:**
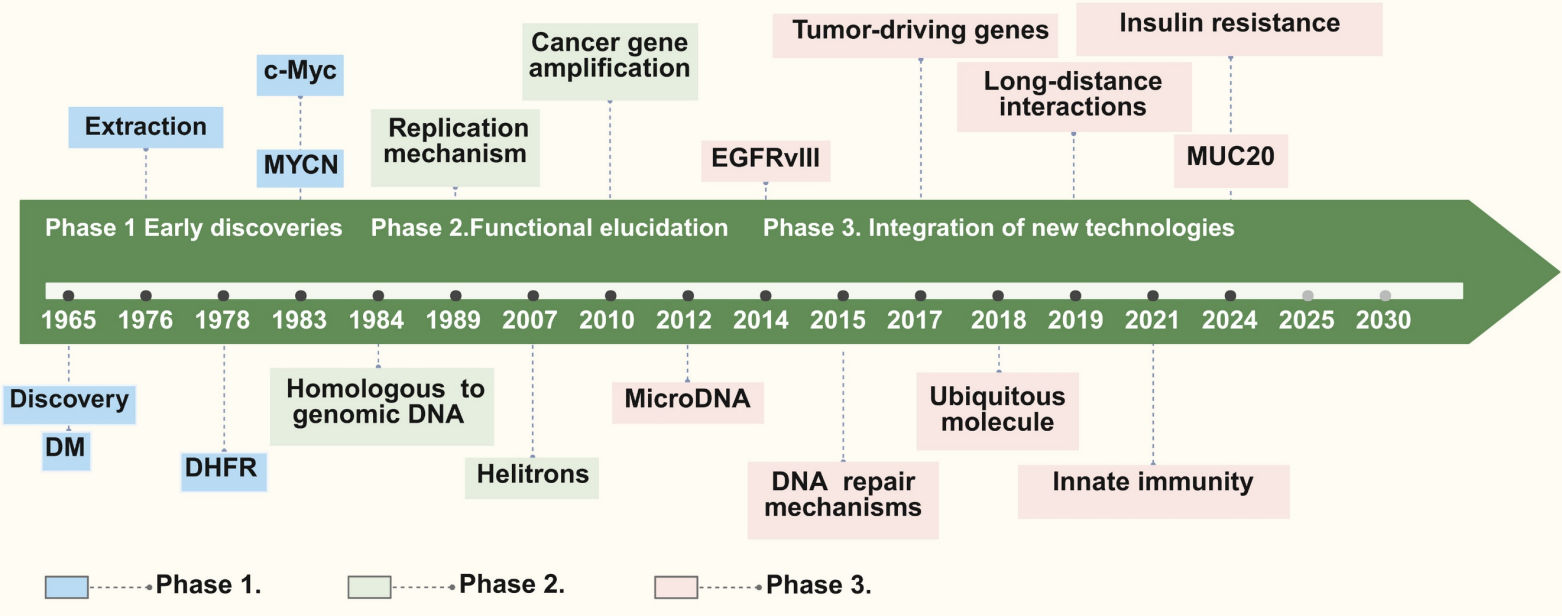
** Discovery history of eccDNA.** The discovery history of eccDNA can be categorized into three primary phases: Phase 1 (Early discoveries), covering discoveries from the identification of double minutes (DM) in 1965 to studies on replication mechanisms in 1989; Phase 2 (Functional elucidation), beginning in 2007 and focusing on topics such as cancer gene amplification, microDNA, and DNA repair mechanisms; and Phase 3 (Integration of new technologies), starting in 2018, which explores the roles of eccDNA in tumor-driving genes, long-distance interactions, innate immunity, and insulin resistance, etc. Key reports are marked along the timeline, with different colors representing each discovery phase.

The exploration of eccDNA spans over five decades, with exploration evolving through distinct phases of discovery, functional characterization, and technological advancements. This progression highlights the growing recognition of significance of eccDNA in both physiological and pathological contexts (**Figure 1**). The division into these three phases is primarily based on the evolution of experimental techniques and the shifting research focus—from initial observations of unusual DNA structures to the elucidation of their biological functions, and finally to high-resolution analyses enabled by modern technologies.

### Early discoveries: laying the foundation

The journey of eccDNA exploration began in 1965 when circular DNA molecules were observed in boar sperm and wheat embryo cells through electron microscopy 6. At the time, these structures were dismissed as rare and biologically insignificant anomalies. Concurrently, “double minutes” (DMs), a peculiar chromosomal structure, were identified in karyotypes of patients with cancer, such as children with embryonic tumors and adults with bronchial cancer 22-23. DMs were the first identified example of eccDNA linked to oncogene amplification, establishing a direct connection between eccDNA and cancer 24. This phase is defined by the initial discovery and descriptive studies using the then-available technology (such as electron microscopy), which led to early insights even though the structures were not fully understood. The focus was on observation and preliminary correlation with disease states rather than functional analysis.

In the late 1970s, the development of cesium chloride (CsCl) gradient centrifugation enabled the isolation and characterization of eccDNA 25. Subsequently, studies in 1978 identified DMs carrying the dihydrofolate reductase (DHFR) gene in methotrexate-resistant tumor cells, demonstrating the role of eccDNA in drug resistance via gene amplification 26. By the 1980s, eccDNA had been implicated in carrying oncogenes such as MYCN 27 and c-Myc 28, highlighting its broader role in tumorigenesis.

### Functional elucidation and expanding horizons

The 1980s and 1990s marked significant advancements in understanding the biological roles of eccDNA. Southern blot analyses confirmed that eccDNA sequences were homologous to chromosomal DNA, suggesting that they originated from specific genomic loci prone to instability 29. Studies demonstrated that eccDNA could replicate independently of the chromosomal genome, underscoring its structural and functional autonomy 21,30,31. These findings solidified the concept that eccDNA was not merely a byproduct of genomic instability but a dynamic genetic entity with potential biological functions. This phase is characterized by the transition from mere observation to functional investigation. The introduction of new molecular biology techniques allowed researchers to begin dissecting the roles of eccDNA in cellular processes, including its involvement in oncogene amplification and drug resistance. The research focus shifted to understanding the origins and replication mechanisms of eccDNA, driven by improved experimental methods.

The emergence of molecular biology techniques has significantly broadened the horizons of eccDNA investigation. For example, eccDNA was found to play a critical role in amplifying oncogenes in cancer, driving tumor heterogeneity, and contributing to therapeutic resistance 21,32-37. These discoveries linked eccDNA to the broader phenomenon of genomic plasticity, emphasizing its adaptive potential in response to cellular stress and environmental challenges.

### Modern era: technological breakthroughs and new insights

The 21st century witnessed a transformative phase in eccDNA investigation, driven by advancements in high-throughput sequencing technologies. These methods enabled the identification of eccDNA with unprecedented resolution, revealing its widespread presence in both normal and tumor tissues 34,35,38-41. The discovery of microDNAs, a subclass of eccDNA typically 200-400 base pairs in size, further expanded the field by uncovering their origins from gene-rich regions such as CpG islands and untranslated regions (UTRs) 1,17,42-43. These smaller eccDNA molecules, often generated through normal genomic processes like transcriptional activity and mismatch repair, highlighted the relevance of eccDNA in non-pathological contexts. This modern phase is defined by the application of cutting-edge genomic and imaging technologies, which have allowed for a detailed and comprehensive mapping of eccDNA. The enhanced resolution and throughput of these methods have not only confirmed earlier findings but have also uncovered new classes of eccDNA and expanded our understanding of its roles beyond cancer, including its participation in normal cellular regulation.

In cancer, eccDNA emerged as a critical driver of oncogene amplification and tumor evolution. For instance, eccDNA carrying amplified copies of oncogenes like EGFR and MYC was implicated in promoting therapeutic resistance and clonal diversity within tumors 18,34,44. Studies also revealed that eccDNA is not restricted to cancerous tissues; it is present in healthy cells, where it may regulate chromatin dynamics and act as a reservoir of regulatory sequences 17,43.

### Emerging applications and future directions

Recent studies have expanded the functional repertoire of eccDNA, uncovering its roles in chromatin remodeling, transcriptional regulation, and immune responses. Notably, small eccDNA molecules have been shown to activate the cGAS-STING pathway, positioning eccDNA as a key mediator of innate immunity during apoptosis and genomic stress 30,44,45. These findings suggest that the functions of eccDNA extend beyond genomic instability, encompassing vital roles in cellular signaling and adaptive responses. Currently, several critical questions remain unanswered: What factors govern the preferential formation of eccDNA from specific genomic regions? How does eccDNA interact with chromosomal DNA and regulatory networks? The integration of single-cell sequencing, advanced imaging technologies, and bioinformatics tools holds great promise for addressing these gaps. At the same time, the development of therapeutic strategies targeting eccDNA—such as inhibitors of eccDNA replication or degradation—could open new avenues for cancer treatment. The discovery history of eccDNA reflects a journey from initial skepticism to the recognition of its central role in genomic plasticity and disease biology. Technological advancements have continually reshaped our understanding of eccDNA, revealing its diverse functions and therapeutic potential. Moving forward, interdisciplinary efforts will be essential in uncovering the full spectrum of roles of eccDNA in health and disease, paving the way for its translation into clinical applications.

## Classification

**Figure 2:**
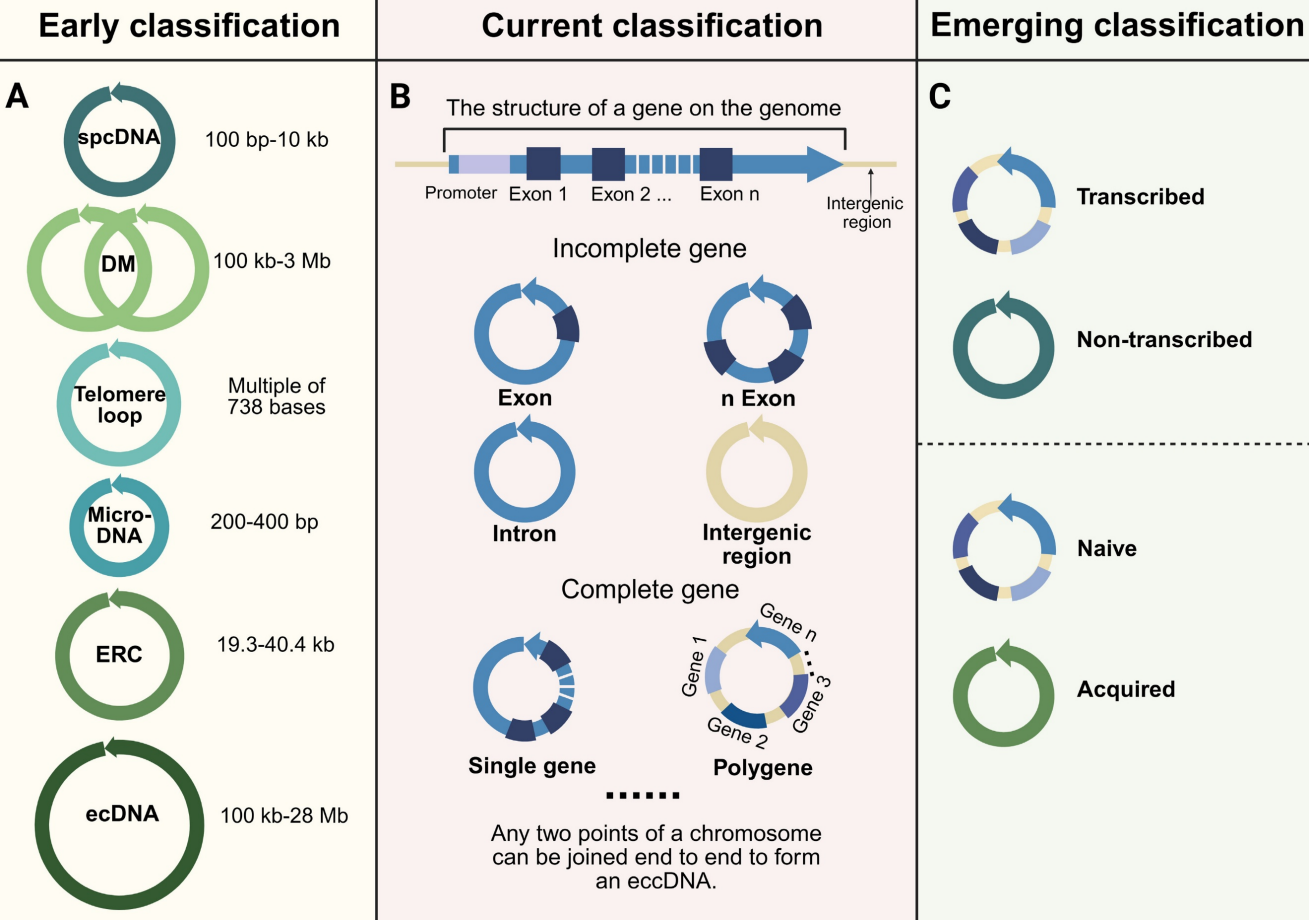
** Classification of eccDNA.** The early classification (A) includes various types of eccDNA such as spcDNA (100 bp-10 kb), double minutes (DM, 100 kb-3 Mb), telomere loops, microDNA (200-400 bp), extrachromosomal rDNA circles (ERC, 19.3-40.4 kb), and ecDNA (100 kb-28 Mb). The current classification (B) categorizes eccDNA based on gene structure, distinguishing between incomplete genes (e.g., exons, introns, intergenic regions) and complete genes (single gene or polygene eccDNA). The emerging classification (C) proposes new categories based on transcriptional activity (transcribed vs. non-transcribed) and origin (naive vs. acquired). The diagram emphasizes that eccDNA can be formed by the joining of any two points on a chromosome.

The categorization of eccDNA has advanced considerably as investigations have enhanced our comprehension of its properties, origins, and roles. (**Figure 2**). To clarify terminology at the outset, eccDNA (extrachromosomal circular DNA) broadly refers to all circular DNA molecules that exist outside chromosomes, including both non-coding and coding elements across various biological contexts. In contrast, ecDNA (extrachromosomal DNA, a subset of eccDNA) specifically describes large, oncogene-carrying circular DNAs frequently found in cancer. The distinction between eccDNA and ecDNA is foundational and has been extensively discussed in recent studies and reviews 24,32,46,47. EcDNA is often megabase-scale and transcriptionally active, while eccDNA encompasses a broader range of smaller and more diverse molecules, many of which are not directly associated with malignancy. Therefore, the use of the terms “early classification,” “current classification,” and “emerging classification” in this review refers not to the chronological obsolescence of any terminology, but rather to the conceptual progression in classification frameworks—from purely structural definitions toward functionally and mechanistically informed schemes. In this context, “early classification” emphasizes historically foundational criteria (such as size and density), “current classification” reflects increased understanding of genomic content, and “emerging classification” highlights functional, regulatory, and pathological distinctions that have gained recent attention.

### Early classification based on size and structure

In early studies, eccDNA and ecDNA were often grouped together due to shared structural features. However, modern perspectives distinguish between these two based on size, content, and function. EcDNA, a cancer-specific subclass of eccDNA, is defined by its large size (100 kb to 28 Mb), frequent oncogene amplification, and contribution to tumor progression 47-49. While "eccDNA" and "ecDNA" are sometimes used interchangeably, recent studies emphasize their functional and clinical distinctions: eccDNA includes all non-chromosomal circular DNAs across biological contexts, whereas ecDNA specifically refers to oncogene-bearing mega-structures driving tumor evolution 24,32,46.

Initially, eccDNA was categorized broadly based on its physical and genomic characteristics (**Figure 2A**). For instance, small polydisperse circular DNA (spcDNA) refers to eccDNA molecules ranging from 100 bp to 10 kb, typically derived from repetitive or intergenic regions of the genome 50-53. In contrast, larger eccDNA molecules, such as double minutes (DMs), a classic example of ecDNA, span from 100 kb to 3 Mb and are most often observed in cancer cells 48,54. DMs have been redefined in contemporary literature as hallmark forms of ecDNA due to their recurrent association with amplified oncogenes and their role in therapy resistance 39,55.

Another early form of eccDNA, telomere loops, consists of telomeric repeats of 738 bases, often resulting from telomere instability or end-to-end chromosomal fusions 56-59. Meanwhile, microDNA—small eccDNA molecules measuring between 200 and 400 bp—originate from gene-rich regions such as transcription start sites or CpG islands. These have been implicated in chromatin remodeling and gene regulation 1,17,42-43. Additionally, extrachromosomal rDNA circles (ERCs), primarily found in yeast and aging cells, are circular DNA molecules derived from rDNA loci that range from 19.3 to 40.4 kb 60-62.

### Current classification based on genomic content

EccDNA can be broadly classified by its genomic content into three subtypes: (1) eccDNA carrying incomplete gene fragments, (2) eccDNA containing full genes, and (3) polygenic eccDNA (i.e., ecDNA) (**Figure 2B**). EccDNA encompasses all extrachromosomal circular DNA molecules, ranging from non-coding fragments to complete gene-containing circular structures, whereas ecDNA refers to large polygenic structures commonly observed in malignancies. Thus, ecDNA represents a specific functional and structural extreme within the eccDNA continuum. Modern classifications of eccDNA have moved beyond size-based categories to consider the genomic content of eccDNA. Specifically, eccDNA encompasses all extrachromosomal circular DNA molecules, ranging from non-coding fragments to complete gene-containing circular structures, whereas ecDNA specifically refers to large polygenic structures commonly observed in malignancies. Recent studies reveal that ecDNA is defined by co-amplification of oncogenes with regulatory enhancers, megabase-scale size (100 kb-28 Mb), and non-Mendelian inheritance patterns, which collectively drive tumor evolution and therapy resistance 24,32,46.

The functional diversity of eccDNA is further reflected in the heterogeneity of its genomic content. Incomplete gene eccDNA includes fragments of genomic regions such as introns, exons, or intergenic sequences 63. While these fragments lack the full coding sequence of a gene, they can still influence transcriptional regulation and chromatin dynamics 13,64. In contrast, complete gene eccDNA molecules encompass entire single genes, enabling their independent transcription and amplification. These molecules can act as self-sufficient units to enhance gene expression under specific conditions 46,65. Larger eccDNA molecules are classified as polygenic eccDNA (i.e., ecDNA), carry multiple genes or gene clusters. These polygenic forms are especially common in cancer, where they amplify oncogenes and associated regulatory elements, driving tumor evolution and adaptive resistance to therapy 33,66.

### Emerging frameworks for functional and pathological classification

Recent developments in role of eccDNA have resulted in the suggestion of classification frameworks that highlight functional and transcriptional attributes. One such framework categorizes eccDNA based on its transcriptional activity, distinguishing between transcribed and non-transcribed eccDNA (**Figure 2C**). Transcribed eccDNA, often enriched with active histone modifications such as H3K27ac, directly amplifies gene expression, particularly in oncogenic contexts 44,46,65,67. Non-transcribed eccDNA, on the other hand, frequently carries intergenic fragments, repetitive sequences (e.g., LINEs, SINEs, and satellite DNA), or truncated genomic elements lacking promoter activity. These non-coding elements may influence chromatin architecture or act as reservoirs of genetic material 10,30,68,69. Some of these fragments are transposons or transposon-derived sequences, such as retrotransposons, which may contribute to genome plasticity and chromatin interactions 70,71. Although not actively transcribed, they may influence chromatin architecture by forming three-dimensional interaction hubs or sequestering chromatin modifiers 72. Notably, some non-transcribed eccDNAs harbor latent regulatory elements such as enhancer-like sequences or transcription factor binding sites, potentially acting as "molecular sponges" to buffer regulatory proteins from their genomic targets 73. Additionally, their role as genetic reservoirs is supported by findings demonstrating reintegration potential during genomic stress events, particularly in contexts of DNA repair deficiency.

Another emerging classification differentiates between naive and acquired eccDNA (**Figure 2C**). These subtypes differ in size, genomic content, functional roles and underlying generation pathways, reflecting their origins in physiological versus pathological contexts. Naive eccDNA typically ranges in size from <100 bp-10 kb and is generated through normal physiological processes, such as DNA repair or chromatin remodeling, and is frequently observed in healthy cells 17,42,74. These circles often carry non-coding genomic regions, including repetitive sequences (e.g., satellite DNA, ribosomal DNA), pseudogenes, or regulatory elements, and are enriched near fragile sites or chromosomal regions prone to replication fork stalling 71. Mechanistically, naive eccDNA formation is linked to microhomology-mediated repair (e.g., during non-homologous end joining or replication slippage) and may arise from the excision of intrachromosomal loops during topoisomerase II activity 75. Naive eccDNA may participate in gene expression regulation and genome stability maintenance in normal cells 76. In contrast, acquired eccDNA is predominantly larger (>100 kb to several megabases) and arises in contexts of genomic instability, such as cancer or chronic inflammation, which arises in pathological conditions, such as cancer or genomic instability, and is often enriched in oncogenes such as EGFR, MYC, and MET, as well as stress-response genes like HSP90 and SOD2, contributing to disease progression and therapeutic resistance 18,47,77-79. Their biogenesis involves catastrophic genomic events, including chromothripsis (chromosomal shattering followed by erroneous repair) and replication-based extrusion of episomes from chromosomal hubs under replication stress 13. Acquired eccDNA may also form via homologous recombination repair (HRR) defects, as seen in BRCA1/2-mutant cancers, where unresolved DNA breaks promote circularization of amplified regions 80. Importantly, acquired eccDNA exhibits transcriptional hyperactivity due to altered chromatin accessibility and enhancer hijacking, driving tumor heterogeneity and adaptation.

It is worth noting that despite increasing interest in functionally oriented eccDNA research, most published studies—continue to describe eccDNA in terms of size (e.g., microDNA, DM), origin (e.g., telomeric circle), or disease context (e.g., oncogene-carrying ecDNA), rather than applying the functional classifications proposed here. This may be due to several reasons. First, many functional and transcriptional properties of eccDNA are still under investigation, and standardized definitions or consensus frameworks for these attributes have not yet been established. Second, experimental pipelines and detection methods are often optimized for size-based enrichment (e.g., for microDNA), leading to reporting bias toward structural rather than functional features. Third, the relatively recent emergence of these classification models has not yet been broadly adopted by the research community, which still relies heavily on historically entrenched descriptors.

Despite these limitations in current usage, the proposed functional and transcriptional classification frameworks offer distinct advantages that address key gaps in existing nomenclature. First, they capture the biological relevance of eccDNA beyond size, including its capacity for transcriptional activity, enhancer hijacking, immune activation, and reintegration potential. Second, this classification aligns more closely with disease mechanisms, distinguishing eccDNAs that drive tumor progression (e.g., transcriptionally active, oncogene-rich eccDNAs) from those involved in physiological maintenance (e.g., naive eccDNAs). Third, function-based classification enables better integration with single-cell transcriptomic and epigenomic data, supporting systems-level analysis of eccDNA behavior under different cellular states. Lastly, as therapeutic interest grows in targeting eccDNA biogenesis or function, a mechanistically grounded classification will facilitate more precise biomarker development and drug targeting. Therefore, we propose this updated classification model as a conceptual scaffold for future research, not to replace existing terminology but to provide a complementary, biologically meaningful lens through which to understand eccDNA diversity.

Together, these classification systems—both historical and modern—provide a comprehensive framework for understanding eccDNA. By integrating structural, genomic, and functional perspectives, researchers can better capture the diversity and complexity of eccDNA. As technological innovations enhance our capacity to identify and analyze eccDNA, forthcoming classifications are likely to further elucidate its functions in both physiological processes and pathological mechanisms. This advancing framework connects fundamental knowledge with clinical applications, emphasizing the roles of eccDNA as both a biomarker for disease detection and a potential therapeutic target. By elucidating these functions, it highlights the promise of eccDNA in improving diagnosis and treatment strategies.

## Comparison between eccDNA and other similar molecules

**Figure 3:**
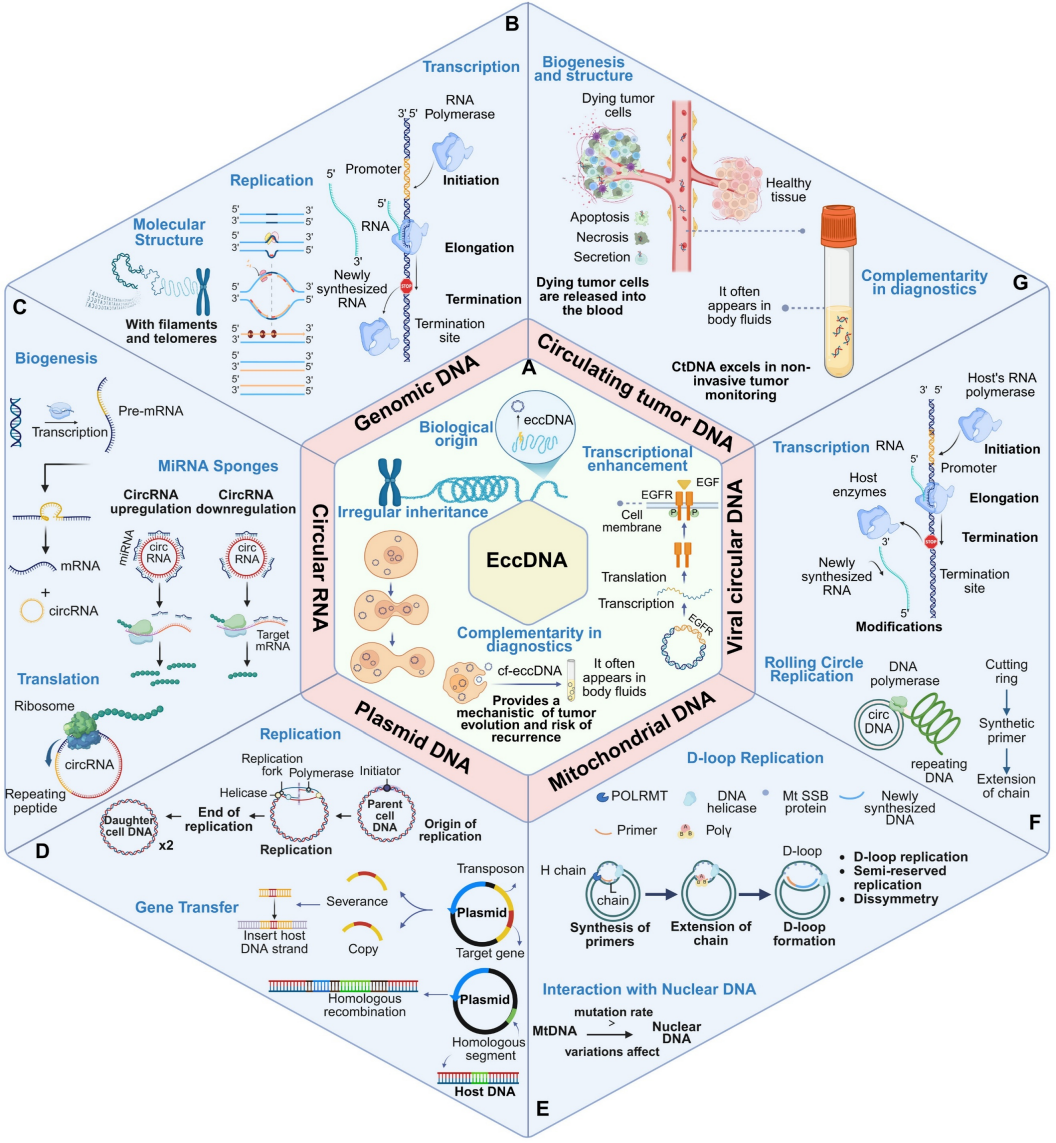
** Comparison of eccDNA with other similar biomacromolecules.** Biomacromolecules, including genomic DNA, circular RNA, plasmid DNA, mitochondrial DNA, viral circular DNA, and circulating tumor DNA, exhibit distinct molecular properties and are involved in a range of critical biological processes. (A) EccDNA originates from genomic DNA and exhibits irregular inheritance. It can enhance transcription, contribute to tumor evolution and recurrence, and appears in body fluids, providing potential for non-invasive diagnostics. (B) Genomic DNA (gDNA) is linear, double-stranded, and contains telomeres. It serves as the primary template for transcription and replication, maintaining genome integrity and transmitting hereditary information. (C) Circular RNA (circRNA) originates from pre-mRNA, functions as a miRNA sponge, regulates gene expression post-transcriptionally, and may be translated into proteins. (D) Plasmids replicate independently via mechanisms like rolling circle replication and can mediate horizontal gene transfer through homologous recombination and transposons. (E) Mitochondrial DNA (mtDNA) replicates via D-loop replication, displays asymmetrical inheritance, and interacts with nuclear DNA; its high mutation rate can affect cellular and organismal functions. (F) Viral circular DNA uses the host's transcription machinery and may undergo post-transcriptional modifications. (G) Circulating tumor DNA (ctDNA) is released from dying tumor cells into body fluids, offering a valuable biomarker for non-invasive tumor monitoring. The panel summarizes key molecular characteristics and biological roles of these circular molecules, including transcription, replication, translation, and interaction with cellular processes, reflecting their complexity and significance in genome regulation, stability, and disease progression.

EccDNA shares structural and functional characteristics with several nucleic acid molecules, including genomic DNA (gDNA), circular RNA (circRNA), plasmid DNA, mitochondrial DNA (mtDNA), viral circular DNA, and circulating tumor DNA (ctDNA). However, these molecules differ markedly in terms of origin, structural configuration, replication mechanisms, biological functions, and disease associations. A detailed comparative analysis along these dimensions can elucidate the unique role of eccDNA in genome dynamics and disease progression (**Figure 3**, **Table 1**).

### Comparison with genomic DNA (gDNA)

#### Structural and functional divergence

Genomic DNA is linear, chromosome-bound, and contains centromeres and telomeres, which are critical for chromosomal stability and faithful segregation during mitosis and meiosis 81,82 (**Figure 3B**). In contrast, eccDNA is circular, lacks centromeric and telomeric sequences, and replicates independently of the chromosomal cycle 68,83 (**Figure 3A**). This independence allows eccDNA to amplify specific genomic regions, particularly those containing oncogenes or regulatory enhancers—under selective pressure.

#### Biological function

While gDNA serves as the static repository of genetic information, eccDNA provides a dynamic genetic reservoir that enables rapid cellular adaptation. EccDNA is often derived from fragile chromosomal regions, including repetitive sequences or oncogene-rich loci such as MYC or EGFR 69,84,85 (**Figure 3A**). Its role in facilitating oncogene amplification, chromatin remodeling, and drug resistance underscores its importance in tumor evolution.

### Comparison with circular RNA (circRNA)

#### Molecular origin

CircRNAs are single-stranded, non-coding RNAs generated through back-splicing of precursor mRNAs, predominantly functioning in post-transcriptional regulation 86,87 (**Figure 3C**). EccDNA, in contrast, originates from genomic DNA via mechanisms such as DNA repair misprocessing, replication stress, or chromothripsis 10,11,88.

#### Function and regulation

CircRNAs often act as miRNA sponges or interact with RNA-binding proteins, influencing gene expression at the RNA level 89 (**Figure 3C**). EccDNA operates primarily at the DNA level, influencing transcription through enhancer activity and gene dosage effects. Unlike circRNA, eccDNA can encode full-length genes and regulatory elements that directly modulate chromatin architecture 13 (**Figure 3A**).

### Comparison with plasmid DNA

#### Contextual differences

Plasmid DNA, commonly found in prokaryotes, facilitates horizontal gene transfer and contributes to traits such as antibiotic resistance 90-93 (**Figure 3D**). Though both plasmids and eccDNA share autonomous replication, eccDNA is intrinsic to eukaryotic genomes and typically functions within a single cell lineage (**Figure 3A**).

#### Replication and evolution

EccDNA enables rapid intratumoral evolution by amplifying oncogenes and bypassing chromatin-based gene regulation. Unlike plasmids, eccDNA contributes to clonal selection and heterogeneity within eukaryotic tumors, particularly under therapeutic stress 11,94 (**Figure 3A**).

### Comparison with mitochondrial DNA (mtDNA)

#### Structural similarity but functional divergence

Both eccDNA and mtDNA are circular and double-stranded (**Figure 3E**). However, mtDNA resides within mitochondria and encodes genes essential for oxidative phosphorylation 95-97, while eccDNA originates from the nuclear genome and is implicated in nuclear gene amplification and adaptive gene regulation 10,68,98 (**Figure 3A**).

#### Stress response

Both molecules exhibit relative resilience to degradation under stress, attributed to their circular configuration. However, their functional outputs differ markedly—mtDNA ensures metabolic homeostasis 99,100, while eccDNA contributes to genome plasticity and tumor adaptability 1,14.

### Comparison with viral circular DNA

#### Origin and oncogenic potential

Viral circular DNA, such as that from HPV or HBV, is exogenous and integrates into the host genome to exploit host replication machinery 101-103 (**Figure 3F**). Although both eccDNA and viral circular DNA are structurally similar and implicated in oncogenesis, their mechanisms differ: viral DNA introduces foreign oncogenes, whereas eccDNA amplifies endogenous oncogenic signals 84,104,105.

#### Functional outcome

Viral DNA may disrupt normal gene regulation through insertional mutagenesis (**Figure 3F**); eccDNA modulates transcriptional activity via enhancer hijacking and gene copy number increase, contributing to tumor heterogeneity 14,106 (**Figure 3A**).

### Comparison with circulating tumor DNA (ctDNA)

#### Biogenesis and structure

EccDNA forms via mechanisms like chromosomal breakage-fusion-bridge (BFB) cycles or chromothripsis and remains nuclear. It often encompasses intact genes or enhancers and is generally larger and more complex than ctDNA 17,24. CtDNA is released into the bloodstream from dying tumor cells and exists as short, fragmented DNA 107,108 (**Figure 3G**).

#### Clinical relevance

CtDNA excels in non-invasive tumor monitoring, enabling real-time profiling of mutational burden and treatment response 109,110. EccDNA, by contrast, contributes to tumor plasticity and drug resistance by amplifying genomic content within the tumor 21.

#### Complementarity in diagnostics

While ctDNA offers insight into existing tumor mutations (**Figure 3G**), eccDNA provides a mechanistic understanding of tumor evolution and recurrence risk (**Figure 3A**). Their combined use may enhance diagnostic precision and therapeutic targeting.

While eccDNA shares structural and functional similarities with other circular and linear DNA molecules, its unique origin, dynamic nature, and role in genomic plasticity set it apart. Ability of eccDNA to amplify gene expression and promote rapid genomic adaptation underlies its significant role in processes like tumor evolution, immune activation, and cellular stress responses. By exploring its relationships with similar molecules, researchers can better understand its distinct biological roles and uncover novel opportunities for therapeutic interventions.

## Formation of eccDNA: a multifaceted process

**Figure 4:**
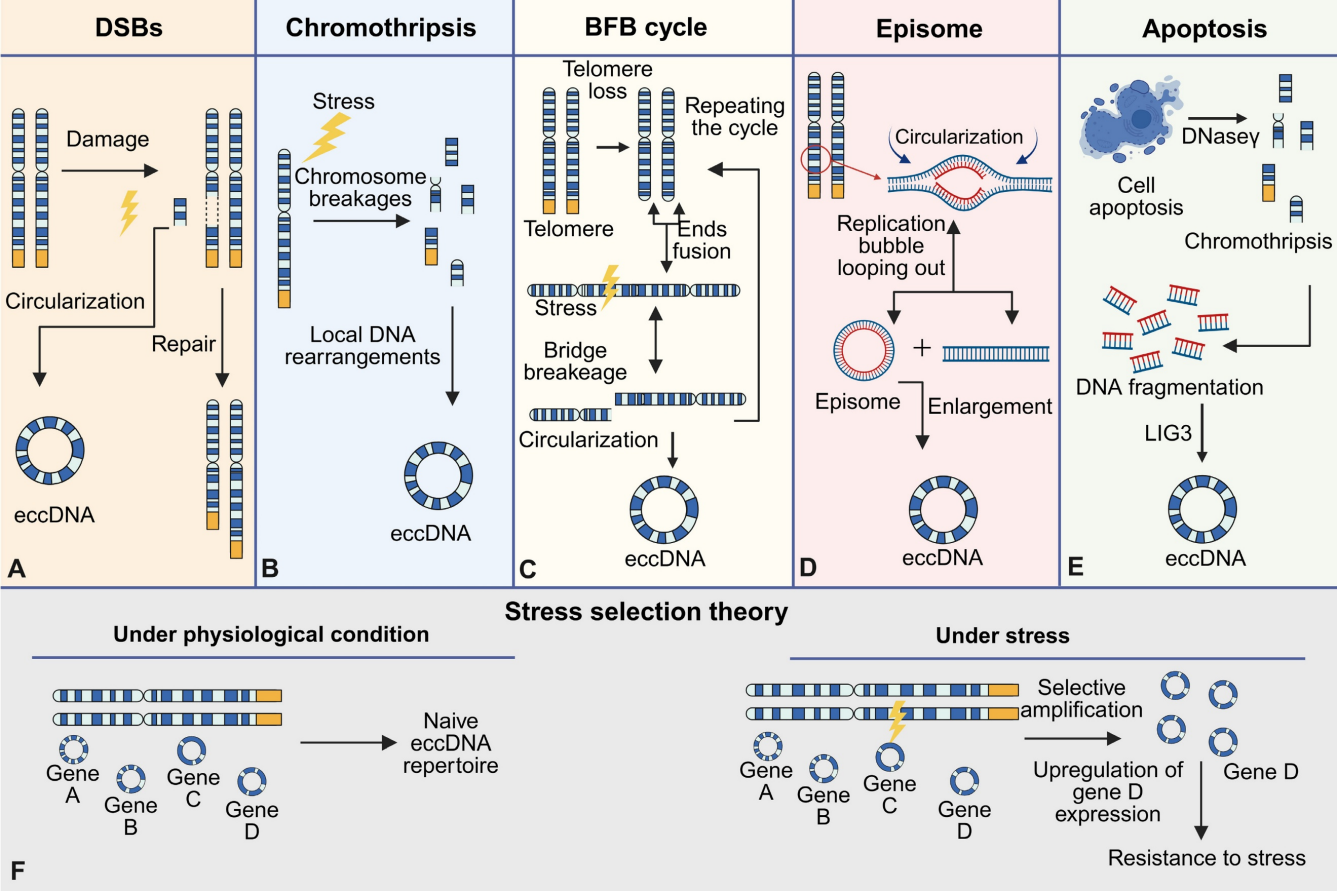
** Mechanisms of eccDNA formation and stress selection theory.** EccDNA formation occurs via multiple distinct mechanisms: (A) Double-strand breaks (DSBs): Chromosomal damage triggers repair pathways that result in the excision and circularization of DNA fragments into eccDNA. (B) Chromothripsis: Under stress conditions, extensive chromosomal fragmentation and localized rearrangements lead to eccDNA generation. (C) Breakage-fusion-bridge (BFB) cycle: Telomere loss induces end-to-end chromosome fusions; subsequent bridge formation and breakage during mitosis produce eccDNA, with the cycle potentially repeating. (D) Episome: During DNA replication, looping out of replication bubbles can generate extrachromosomal DNA that circularizes to form episomes, which may further expand into eccDNA. (E) Apoptosis: Programmed cell death involves DNase-mediated fragmentation of chromatin, with ligation of DNA fragments (e.g., via LIG3) forming eccDNA; chromothripsis may also occur in this context. (F) Stress selection theory: Under physiological conditions, a basal, naive eccDNA repertoire is maintained. Upon exposure to stress, selective amplification of stress-responsive eccDNAs (for example, containing gene D) leads to increased gene expression and promotes cellular resistance to stress.

The formation of eccDNA is a complex process driven by genomic instability, cellular stress responses, and DNA repair mechanisms (**Figure 4**). It has been increasingly recognized as a result of diverse molecular pathways, reflecting both pathological disruptions and normal physiological processes. In this context, eccDNA can be broadly categorized into naive eccDNA and acquired eccDNA. Naive eccDNA—also referred to as physiological eccDNA—typically measures less than a few hundred base pairs, often termed microDNAs, and originates from CpG islands, gene-dense loci, and transcription start sites 17,42,51. These molecules are believed to participate in genomic regulation and chromatin remodeling. In contrast, acquired eccDNA is often larger (ranging from 0.5 to 50 kb), functionally enriched with oncogenes or stress-response genes, and is formed under conditions of genomic stress such as replication stress, apoptosis, or exposure to genotoxic agents 30,63,84,85.

One of the key events initiating eccDNA formation is double-strand breaks (DSBs), which occur during replication stress, chromosomal rearrangements, or exposure to genotoxic agents. The repair of these breaks by non-homologous end joining (NHEJ) or homologous recombination (HR) can result in the excision of specific DNA fragments 10,111. These excised fragments, often derived from repetitive regions or fragile sites, circularize to form eccDNA. The high prevalence of repetitive sequences, such as satellite DNA or gene-rich loci, within eccDNA highlights the susceptibility of these regions to errors during DNA replication and repair 112-114. Furthermore, the widely accepted mechanistic models underlying eccDNA biogenesis encompass the breakage-fusion-bridge (BFB) cycle, chromothripsis (chromosomal shattering), episome-based excision, and translocation-deletion-amplification processes 15,31,115.

### DNA damage repair

The biogenesis of eccDNA is intrinsically linked to chromosomal DNA damage, functional aberrations, and subsequent repair processes, involving dynamic interplay of multiple molecular pathways 116. Central to this phenomenon are double-strand breaks (DSBs), which drive eccDNA formation through chromosomal fragmentation and subsequent circularization (**Figure 4A**). DSBs commonly arise during mitotic/meiotic replication or recombination, while exogenous stressors such as radiation and genotoxic agents exacerbate DSB frequency or repair errors, thereby amplifying eccDNA accumulation 42,116-118. Mechanistically, DSB repair pathways play pivotal roles: The non-homologous end joining (NHEJ) pathway facilitates direct ligation and circularization of DNA ends via KU70/80 complex-mediated recruitment of core repair proteins (e.g., XRCC4, LIG4) 119-121. In parallel, microhomology-mediated end joining (MMEJ) bridges resection gaps by annealing 2-25 bp microhomologous sequences flanking DSBs 122,123. Notably, approximately 66.36% of eccDNA molecules exhibit 4-18 bp direct repeats near junction sites, strongly supporting MMEJ as a dominant mechanism underlying eccDNA biogenesis 75,122,124. Homologous recombination (HR) further contributes to eccDNA generation in regions with high sequence homology, where template-driven repair may inadvertently release circularized DNA fragments 17,31. Compared to DSB repair pathways, single-strand break repair (SSBR) pathways, such as mismatch repair, have a higher success rate 125. Although SSBR systems demonstrate higher fidelity, their engagement in eccDNA formation is implicated through microhomology-directed circularization of single-stranded DNA intermediates, particularly at regions enriched with direct repeats 71,125. This process depends on the activity of DNA ligase 3 (LIG3), which promotes the circularization and closure of DNA ends by catalyzing the ligation of single-strand breaks 71,125.

Importantly, apoptosis has also emerged as a critical mechanism in the generation of acquired eccDNA, particularly under pathological conditions. Caspase-dependent nucleases, such as DNase γ, are activated during programmed cell death and cleave chromosomal DNA at internucleosomal sites, generating short DNA fragments. These fragments undergo circularization facilitated by LIG3 via microhomology-mediated end joining, resulting in eccDNA with a size distribution typically ranging from 0.5 to 50 kb 30. This mechanism aligns closely with the biogenesis of acquired eccDNA, which often arises in response to cellular stress or damage. Collectively, these findings underscore eccDNA biogenesis as a multifaceted outcome of genomic stress and repair pathway crosstalk, offering critical insights into the molecular basis of genome instability.

### Chromothripsis, breakage-fusion-bridge cycles, and other pathological drivers

In addition to DSB repair, chromothripsis, a catastrophic genomic event characterized by the fragmentation and chaotic reassembly of chromosomes, is another well-documented mechanism for eccDNA biogenesis. During chromothripsis, some DNA fragments are circularized rather than reintegrated into the genome, forming eccDNA (**Figure 4B**). This process is particularly prominent in cancer cells, where eccDNA is enriched with oncogenes and other regulatory elements that drive tumorigenesis 77,126. The BFB cycle, a hallmark of telomere dysfunction, also contributes to eccDNA formation. Chromosomal ends lacking telomeres undergo end-to-end fusions, forming unstable dicentric chromosomes that break during mitosis (**Figure 4C**). These chromosomal fragments can subsequently circularize, further amplifying genomic instability and gene copy number variation 13,127. Besides, the episome model, a classic mechanism of eccDNA biogenesis, involves the generation of eccDNA through DNA slippage and R-loop formation during synthesis (**Figure 4D**). Small chromosomal fragments can fuse, recombine, and undergo circular amplication to form eccDNA 21,46,67. Alternatively, these fragments can integrate into chromosomes via BFB cycles, creating homologous stained regions (HSR) with gene amplification potential 128,129.

### Cellular apoptosis and other pathological drivers

Apoptosis contributes significantly to the formation of acquired eccDNA, especially in stressed or damaged cells. Previous studies have shown a significant positive correlation between the apoptotic process and the biogenesis of eccDNA 30. Mechanistic investigations reveal that eccDNA generation in apoptotic cells relies on caspase-dependent nucleases (e.g., DNase γ) -mediated chromosomal DNA fragmentation, as well as catalytic activity of LIG3 to complete circularization (**Figure 4E**). During this process, DNase γ generates single-stranded DNA breaks by cleaving internucleosomal linker regions, while LIG3 facilitates the aberrant joining of DNA ends through microhomology-mediated ligation, ultimately producing eccDNA molecules with a multimodal size distribution (0.5-50 kb) 30. These findings indicate that apoptosis-associated genomic fragmentation events can be reshaped into structural eccDNA via LIG3-dependent circularization mechanisms.

Cellular stress further amplifies the production of eccDNA, particularly in cancer and other disease states. Oncogene overexpression, replication stress, and exposure to genotoxic agents such as chemotherapeutic drugs have been shown to increase eccDNA formation 84,130,131. This adaptive response may enable cells to amplify stress-response genes or oncogenes, facilitating survival under adverse conditions. For instance, eccDNA carrying amplified oncogenes such as MYC or EGFR contributes to tumor progression, clonal heterogeneity, and resistance to targeted therapies 63,84,85. Such stress-induced eccDNA, often classified as acquired eccDNA, demonstrates distinct functional roles in supporting tumor adaptability and therapeutic resistance. These features underline the dual role of eccDNA not only as a marker but also a mediator of cellular adaptation in response to genomic stress.

### Physiological mechanisms and adaptive functions

Emerging evidence suggests that eccDNA formation is not restricted to pathological contexts but also occurs as a normal physiological process. For example, during routine genomic maintenance, excision of extraneous or damaged DNA fragments may result in the generation of smaller eccDNA molecules. These physiological eccDNA molecules, often referred to as microDNAs, are typically less than a few hundred base pairs in size and arise from gene-rich regions, CpG islands, and transcription start sites 17,42,51,132. Unlike eccDNA generated under stress, physiological eccDNA is thought to play a role in chromatin remodeling and genomic regulation rather than acting as a driver of genomic instability.

The "stress selection theory" proposes that eccDNA formation is not entirely stochastic but involves a selective process whereby cells preferentially retain eccDNA molecules that confer adaptive advantages 20. This theory predominantly pertains to acquired eccDNA, which often harbors oncogenes, enhancers, or regulatory sequences, thereby promoting evolutionary fitness in tumor cells (**Figure 4F**). Stress-induced eccDNA formation underscores its role as a reservoir of genomic plasticity, allowing cells to rapidly adapt to environmental and therapeutic pressures.

### Interplay between eccDNA formation mechanisms

Although multiple pathways leading to eccDNA formation have been described independently, recent evidence suggests that these mechanisms often act in concert, forming a mechanistic continuum rather than isolated events. For instance, DSBs, which represent a common initial trigger of eccDNA biogenesis, may originate from replication stress, genotoxic insults, or telomere dysfunction 111,112,131. These DSBs frequently generate fragmented chromosomal regions that, rather than being precisely repaired, may be processed into circular DNA via multiple repair routes.

A particularly important interaction exists between DSB repair pathways and BFB cycle. BFB cycles are initiated by telomere erosion or dysfunction, leading to end-to-end chromosomal fusion. These dicentric chromosomes are unstable during mitosis and often undergo breakage, producing highly damaged chromosomal fragments 127,133. These fragments are then subject to error-prone repair via mechanisms such as NHEJ or MMEJ, which in turn promote the circularization of DNA and eccDNA formation 35,134. Thus, BFB cycles not only reflect chromosomal instability but actively contribute to eccDNA production by feeding the DSB repair system with eccDNA-prone substrates.

Similarly, chromothripsis—a catastrophic chromosomal shattering event—generates extensive genomic fragmentation within a confined nuclear region 77,135. These shattered fragments may follow different fates: some are re-integrated into the genome through complex rearrangements, while others evade reintegration and circularize to form eccDNAs. Chromothripsis-derived eccDNAs often exhibit high heterogeneity in size and content and are enriched in repetitive or oncogene-rich loci 77,126. Interestingly, these fragments may also engage BFB cycles if reintegration fails or occurs at unstable chromosomal ends, further amplifying the probability of eccDNA generation.

Additionally, DSBs and chromothripsis can create substrates suitable for episome-based extrusion 77,136,137, in which excised genomic loops—such as those containing enhancers, promoters, or replication origins—are released and circularized through topoisomerase activity or DNA ligase-mediated closure 18,134,136. These episomes may undergo subsequent rolling-circle amplification, particularly under replication stress or checkpoint failure, creating high-copy eccDNA structures similar to those seen in cancer-associated ecDNA 47,63.

Together, these findings suggest that eccDNA biogenesis is rarely a linear, single-pathway process. Instead, it involves dynamic crosstalk between chromosomal instability events, DNA repair pathways, and structural genome remodeling processes. The cooperative action of BFB cycles, chromothripsis, and DSB repair not only increases the likelihood of eccDNA formation but also shapes the molecular features and functional properties of the resulting eccDNAs. This complexity is reflected in the coexistence of naive and acquired eccDNA populations, each shaped by specific triggers, sizes, genomic origins, and biological roles. This integrative perspective may help explain the heterogeneity and context specificity of eccDNA observed across different cell types and disease states 10, 47,131.

### Future perspectives on eccDNA biogenesis

Despite significant advances in understanding the biogenesis of eccDNA, many questions remain unresolved. The sequence specificity of eccDNA formation, such as why certain genomic regions are more prone to excision and circularization, requires further investigation. Similarly, the regulatory mechanisms governing the stability, inheritance, and cellular distribution of eccDNA remain poorly understood. Advances in single-cell sequencing and imaging technologies are expected to address these gaps, providing deeper insights into the molecular pathways driving eccDNA biogenesis and its functional implications. The formation of eccDNA reflects the interplay between genomic instability, stress responses, and repair mechanisms, showcasing its roles in both pathological and physiological contexts. By integrating classical models such as DSB repair and chromothripsis with emerging theories of stress-induced and adaptive eccDNA production, researchers can uncover its broader significance in health and disease. Further elucidation of the molecular processes underlying eccDNA formation will pave the way for novel diagnostic and therapeutic strategies targeting its diverse functions.

## Investigative methods

**Figure 5:**
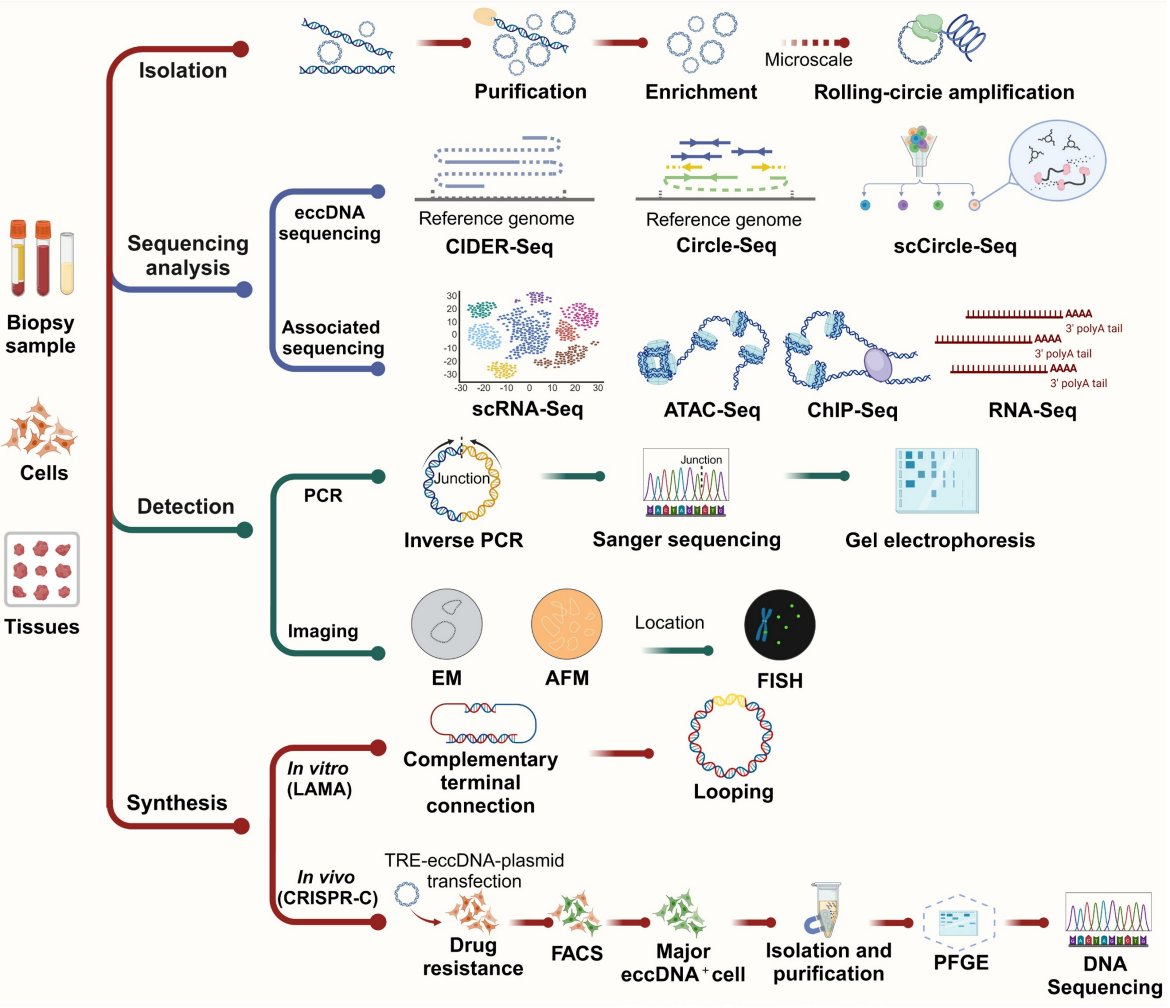
** Investigative methods for eccDNA.** Biopsy samples, cells and tissues first undergo eccDNA isolation, purification, and—where needed-microscale rolling-circle amplification. Both long-read (e.g., CIDER-Seq) and short-read (Circle-Seq and scCircle-Seq) strategies enable sequence determination, supported by bioinformatics pipelines and complementary analyses (e.g., ChIP-Seq, ATAC-Seq, RNA-Seq, scRNA-Seq). Validation includes imaging (EM, AFM) and location analysis (FISH), while junctions can be resolved by inverse PCR and Sanger sequencing. Synthetic generation of eccDNA *in vitro* (LAMA) or *in vivo* (CRISPR-C) allows functional studies in cell lines following plasmid transfection, drug selection, and FACS sorting. Subsequent purification steps (including PFGE) prepare eccDNA for final DNA sequencing and detailed downstream analyses.

The investigation of eccDNA has advanced rapidly, driven by the development of diverse and increasingly sophisticated methodologies for its isolation, characterization, and functional analysis. These methods—ranging from classical biochemical techniques to next-generation sequencing (NGS) and single-cell resolution technologies—are essential for elucidating the biogenesis, structure, distribution, and roles of eccDNA in both physiological and pathological contexts. In this section, we highlight key methodologies used in eccDNA research, evaluating their applications, mechanisms, and limitations (**Figure 5**).

### Isolation and enrichment of eccDNA

The isolation of eccDNA is a critical first step for downstream analyses. Early studies used density gradient centrifugation with cesium chloride (CsCl) to exploit the unique buoyant density of circular DNA 1,9,138. While effective, this method is labor-intensive and unsuitable for high-throughput applications. Contemporary protocols commonly use exonuclease treatments to degrade linear genomic DNA while enriching circular DNA, which is resistant to such digestion 139-141. This approach remains a foundational step in most eccDNA studies due to its operational simplicity and compatibility with sequencing workflows.

### High-throughput sequencing and bioinformatics analysis

#### Short-read vs. long-read sequencing in eccDNA studies

The integration of sequencing technologies into eccDNA research has revolutionized the field. Short-read sequencing (e.g., Illumina platforms) offers high base accuracy and throughput, making it ideal for detecting small eccDNA and high-resolution mapping of eccDNA breakpoints 67,134. However, its short read length limits its ability to resolve complex or repetitive eccDNA structures.

Long-read sequencing technologies, such as PacBio SMRT and Oxford Nanopore, provide extended reads that enable the identification of large or complex eccDNAs and their structural features with minimal assembly 68,73,141. These platforms are particularly advantageous in detecting eccDNAs containing repetitive elements or structural rearrangements. Nonetheless, they may suffer from higher per-read error rates and require more extensive computational correction and validation.

#### Single-cell eccDNA sequencing methods

The development of single-cell sequencing tools has opened new avenues for understanding eccDNA heterogeneity at the cellular level. Notably, scCircle-Seq combines exonuclease digestion with whole genome amplification and NGS, allowing for the profiling of eccDNA within individual cells 71. This method is particularly powerful for studying tumor heterogeneity, lineage tracing, and eccDNA-driven clonal evolution. Complementary methods, such as scATAC-Seq and scRNA-Seq, help map chromatin accessibility and transcriptional consequences of eccDNA, respectively, in single cells 71,142.

#### Bioinformatics tools and databases

Advanced computational pipelines are critical for distinguishing genuine eccDNA from sequencing artifacts. Tools such as ecc_finder, Circle_finder, and AmpliconArchitect 68,143 analyze split-reads, discordant read pairs, and circular amplification signatures to identify eccDNA from bulk or single-cell sequencing data. These tools enable the mapping of eccDNA to its genomic loci of origin, as well as the identification of functional elements like promoters, enhancers, and exons. However, the dependence on computational pipelines introduces challenges related to false positives and detection sensitivity, underscoring the importance of careful data validation 68,144.

Public databases such as CircleBase (http://circlebase.maolab.org), eccDNAdb (http://www.eccdnadb.org) and eccDNA Atlas (https://lcbb.swjtu.edu.cn/eccDNAatlas) provide curated eccDNA annotations, associated genes, and disease links, facilitating cross-study comparisons and hypothesis generation. These resources are increasingly vital for validating newly detected eccDNAs and integrating them into functional networks 105,145,146.

### Detection of eccDNA

Microscopy-based methods continue to be valuable tools for visualizing eccDNA. Early use of electron microscopy (EM) by Hotta and Bassel (1965) confirmed the circular topology of eccDNA 6. Optical microscopy (OM), while useful for observing larger eccDNA species such as double minutes (DMs), lacks sufficient resolution for small eccDNAs. Recent innovations, including super-resolution 3D structured illumination microscopy (3D-SIM) 147 and scanning/transmission electron microscopy (SEM/TEM) 148,149, often combined with fluorescence *in situ* hybridization (FISH) 150, enable precise localization and dynamic tracking of eccDNA molecules.

For detection, traditional methods such as PCR combined with gel electrophoresis and Sanger sequencing remain widely used for validation and size estimation 68,151,152.

### Synthesis of eccDNA: LAMA and CRISPR-C

Two prominent *in vitro* approaches for eccDNA synthesis are LAMA (ligase-assisted minicircle accumulation) and CRISPR-C. LAMA generates minicircles by annealing and ligating complementary oligonucleotides or linear DNA fragments. Recent advancements have streamlined the process by reducing oligo complexity and increasing yield efficiency 153. This method is especially useful for producing synthetic eccDNA constructs for reporter assays or structure-function studies.

CRISPR-C utilizes targeted Cas9-mediated cleavage followed by cellular repair mechanisms that circularize excised DNA fragments. This approach enables the generation of long and specific eccDNAs, which can be used to mimic endogenous eccDNA formation or introduce regulatory elements for functional assays 154.

### CRISPRi as a future tool in eccDNA research

An emerging tool for functional studies is CRISPR interference (CRISPRi), which uses catalytically inactive dCas9 fused with transcriptional repressors to modulate gene activity without altering the DNA sequence 146. CRISPRi can be applied to suppress genes involved in eccDNA biogenesis, allowing researchers to dissect regulatory pathways without inducing double-stranded breaks 155. It also presents opportunities for synthetic modulation of eccDNA-bearing oncogenes in therapeutic contexts. Further development and validation of CRISPRi platforms in eccDNA-focused applications are highly encouraged.

### Functional studies and experimental models

To determine the functional impact of eccDNA, researchers use a variety of *in vitro* and *in vivo* models. Cultured cancer cell lines, such as glioblastoma or neuroblastoma models, allow for perturbation studies of eccDNA-related oncogenes 143,156,157. CRISPR-Cas9 systems are frequently employed to artificially induce eccDNA formation for mechanistic studies 71,158. Coupled with ChIP-Seq, researchers can assess histone modification patterns (e.g., H3K27ac) on eccDNA, reflecting their transcriptional activity 10. RNA-Seq further reveals transcriptional consequences of eccDNA formation or amplification.

Animal models, particularly genetically engineered mice, provide vital insights into the *in vivo* behavior of eccDNA. These models have been used to study roles of eccDNA in tumor evolution, therapy resistance, and clonal selection 84,159. Emerging integration of lineage tracing and spatial transcriptomics with eccDNA analyses will likely enhance our understanding of eccDNA-driven biological complexity in tissues.

The study of eccDNA benefits from an integrative methodological approach, combining classical biochemical tools, high-throughput and single-cell sequencing, imaging modalities, and gene-editing platforms. Recent progress in long-read sequencing, single-cell resolution techniques, and *in vitro* synthesis methods has greatly expanded the research toolkit. Furthermore, computational platforms and curated databases now play a central role in standardizing and interpreting eccDNA data. Looking ahead, the implementation of CRISPRi and real-time imaging techniques promises to further illuminate the complex regulatory networks involving eccDNA, with implications for diagnostics, cancer biology, and gene regulation research.

## Biological functions

**Figure 6:**
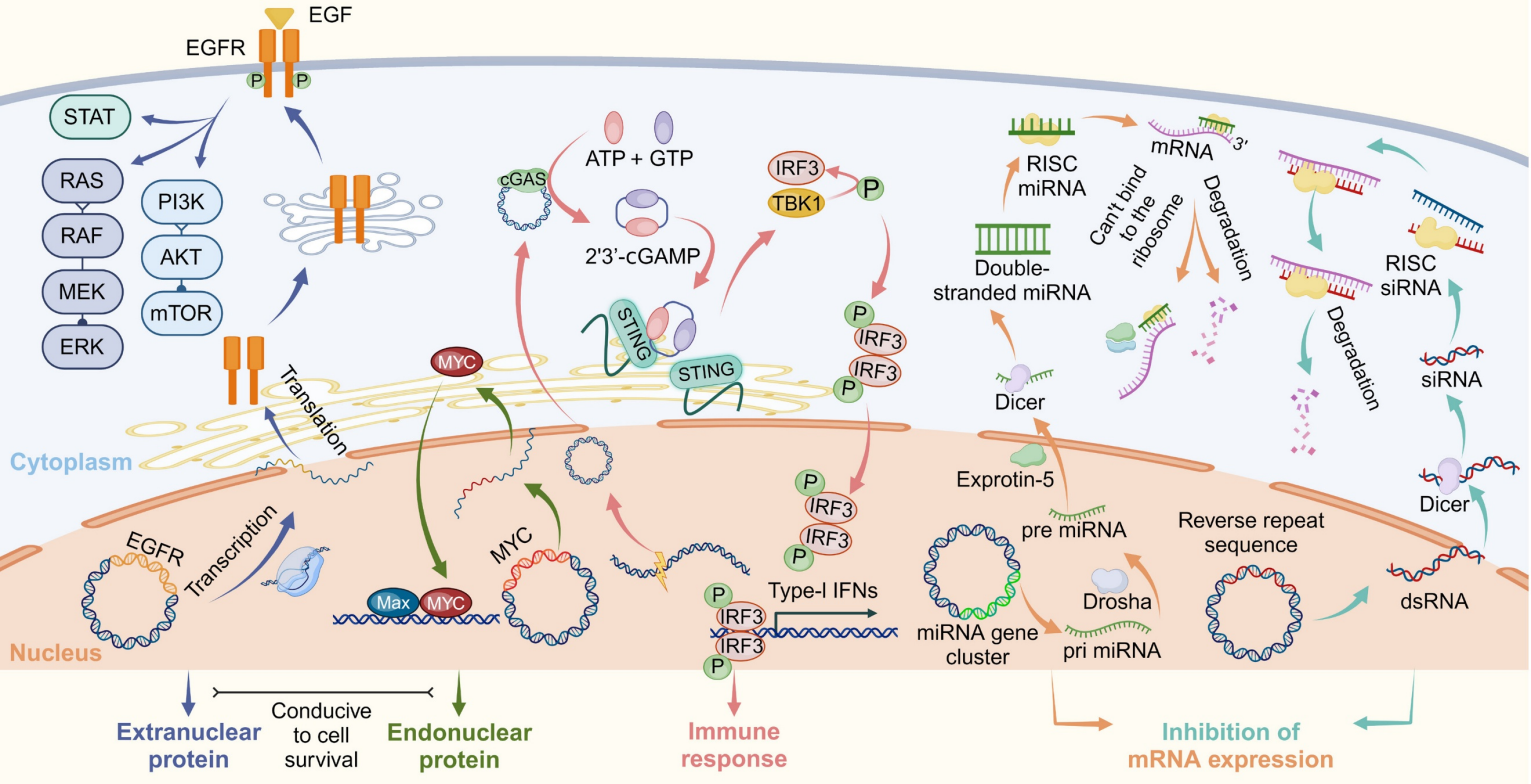
** Representative biological functions of eccDNA.** EccDNA regulates diverse cellular processes through transcriptional activation, immune signaling, and RNA interference. In the nucleus, eccDNA can drive expression of oncogenes such as EGFR and MYC, promoting the production of extranuclear and endonuclear proteins that support cell survival and proliferation. eccDNA may also encode microRNA (miRNA) clusters, which are processed by Drosha and Dicer into mature miRNAs. These miRNAs are incorporated into the RNA-induced silencing complex (RISC) to mediate mRNA degradation or translational repression. In the cytoplasm, eccDNA activates the cGAS-STING pathway. Detection by cGAS triggers synthesis of 2′3′-cGAMP, which activates STING and downstream phosphorylation of TBK1 and IRF3. Phosphorylated IRF3 translocates to the nucleus to induce type I interferon (IFN) expression, initiating immune responses. Additionally, reverse repeat sequences in eccDNA can form double-stranded RNA (dsRNA), which is processed into siRNAs that promote mRNA silencing via RISC. These mechanisms highlight the dual roles of eccDNA in promoting oncogenic signaling and regulating immune defense and gene expression.

EccDNA exhibits a wide array of biological functions, ranging from transcriptional regulation to immune system activation and genomic adaptability. These functions highlight its importance in both normal physiological processes and disease pathogenesis, particularly in cancer. Below, we explore its roles in transcriptional amplification, immune response, and its broader implications for genomic plasticity and disease (**Figure 6**).

### Transcriptional amplification and gene regulation

The structural features of eccDNA, including its ability to carry replication origins, promoters, enhancers, and intact genes, make it a potent amplifier of transcriptional activity. In cancer, eccDNA often carries oncogenes such as MYC, EGFR, and CDK4, enabling their overexpression and contributing to tumor progression 20,63,160. Unlike chromosomal DNA, eccDNA exists in a more open chromatin state, enriched with transcriptionally active histone marks like H3K27ac, which facilitate robust gene transcription 44,45,160. This characteristic allows eccDNA to bypass normal chromatin regulatory mechanisms, leading to enhanced transcriptional output and increased oncogenic signaling.

Moreover, eccDNA can modulate transcription beyond oncogenes. For instance, eccDNA carrying stress-response genes has been shown to enhance cellular survival under challenging conditions, such as genotoxic stress or nutrient deprivation 10,64,160. In rapidly dividing cells, eccDNA containing histone gene clusters can stabilize chromatin by compensating for deficiencies in histone protein production, thereby maintaining genomic integrity 10,68. Additionally, small eccDNA-derived RNAs, such as microRNAs (miRNAs) and small interfering RNAs (siRNAs), play a post-transcriptional regulatory role, influencing broader gene expression networks and cellular behavior 43,71,104. Specifically, eccDNA-derived miRNAs can bind to complementary sequences in the 3′-untranslated region (3′-UTR) of target mRNAs, leading to translational repression or degradation of these transcripts, thereby fine-tuning gene expression programs critical for tumor growth and metastasis 43,161. Conversely, eccDNA-generated siRNAs may integrate into RNA-induced silencing complexes (RISCs), guiding sequence-specific cleavage of homologous mRNAs to silence oncogene suppressors or pro-apoptotic factors, further amplifying cancer cell survival 162,163. These RNA-mediated regulatory networks extend the functional repertoire of eccDNA beyond DNA-level amplification, enabling dynamic control of cellular behavior.

### Immune activation and non-transcriptional roles

EccDNA plays a crucial role not only in transcription but also in activating the innate immune system. When small eccDNA molecules are released into the cytoplasm during DNA damage or apoptosis, they serve as potent activators of the cGAS-STING pathway, triggering immune responses and inflammation 30. These molecules often contain repetitive sequences and are found in abundance in stressed or damaged cells 30. The activation of cGAS-STING by eccDNA links it to the cellular stress response, positioning eccDNA as an essential mediator in immune surveillance.

Furthermore, eccDNA has been found in extracellular vesicles, such as exosomes, where it may play a role in intercellular communication 164. Though this function is still under investigation, eccDNA-containing vesicles may facilitate the horizontal transfer of genetic material, influencing the behavior of recipient cells. This highlights the dual function of eccDNA as both a marker of genomic instability and an active participant in cellular signaling processes.

### Broader implications for genomic plasticity and disease

EccDNA plays a crucial role in genomic flexibility due to its unique characteristics. Acting as an independent reservoir of genetic material, it enables cells to adapt quickly to environmental pressures, including those arising from therapeutic treatments. This ability to adapt is particularly noticeable in cancer, where eccDNA contributes to tumor evolution, clonal diversity, and drug resistance. For instance, in glioblastoma, eccDNA containing the EGFRvIII mutation has been shown to directly contribute to resistance against targeted therapies 10,47.

Beyond cancer, eccDNA is gaining attention as a potential biomarker for early disease detection and prognosis. Its stable circular structure and presence in bodily fluids such as blood and urine make it an appealing candidate for liquid biopsy applications 7,83,124,130,165. Additionally, certain eccDNA signatures have been linked to a variety of diseases, including cardiovascular conditions and pregnancy-related disorders, highlighting its potential for diagnostic use 124,166,167.

### Implications of the stress selection theory

The stress selection theory provides a conceptual framework that integrates eccDNA formation with cellular adaptation under selective pressure. In cancer, cells exposed to genotoxic stressors—including chemotherapy, radiation, or oxidative damage—are more likely to accumulate and retain eccDNAs that harbor amplified oncogenes, drug resistance elements, or stress-response regulators such as HSP90, EGFR*,* and MYC. Recent studies have shown that treatment with DNA-damaging agents can markedly elevate the production of eccDNA *in vitro* and *in vivo*, and the resulting eccDNA is often transcriptionally active, promoting resistance and clonal outgrowth 17,20. This suggests a non-random, evolutionarily advantageous selection of functional eccDNAs under therapeutic stress. In a broader biological context, the theory also explains eccDNA enrichment in immune activation, senescence, and aging tissues, where genomic instability and selective pressure shape eccDNA landscapes. Future studies using lineage tracing, CRISPR-based deletion of eccDNA hotspots, and real-time single-cell imaging will be instrumental in validating this theory and uncovering its full regulatory impact.

## Distribution of eccDNA in human tissues with different disease conditions

**Figure 7:**
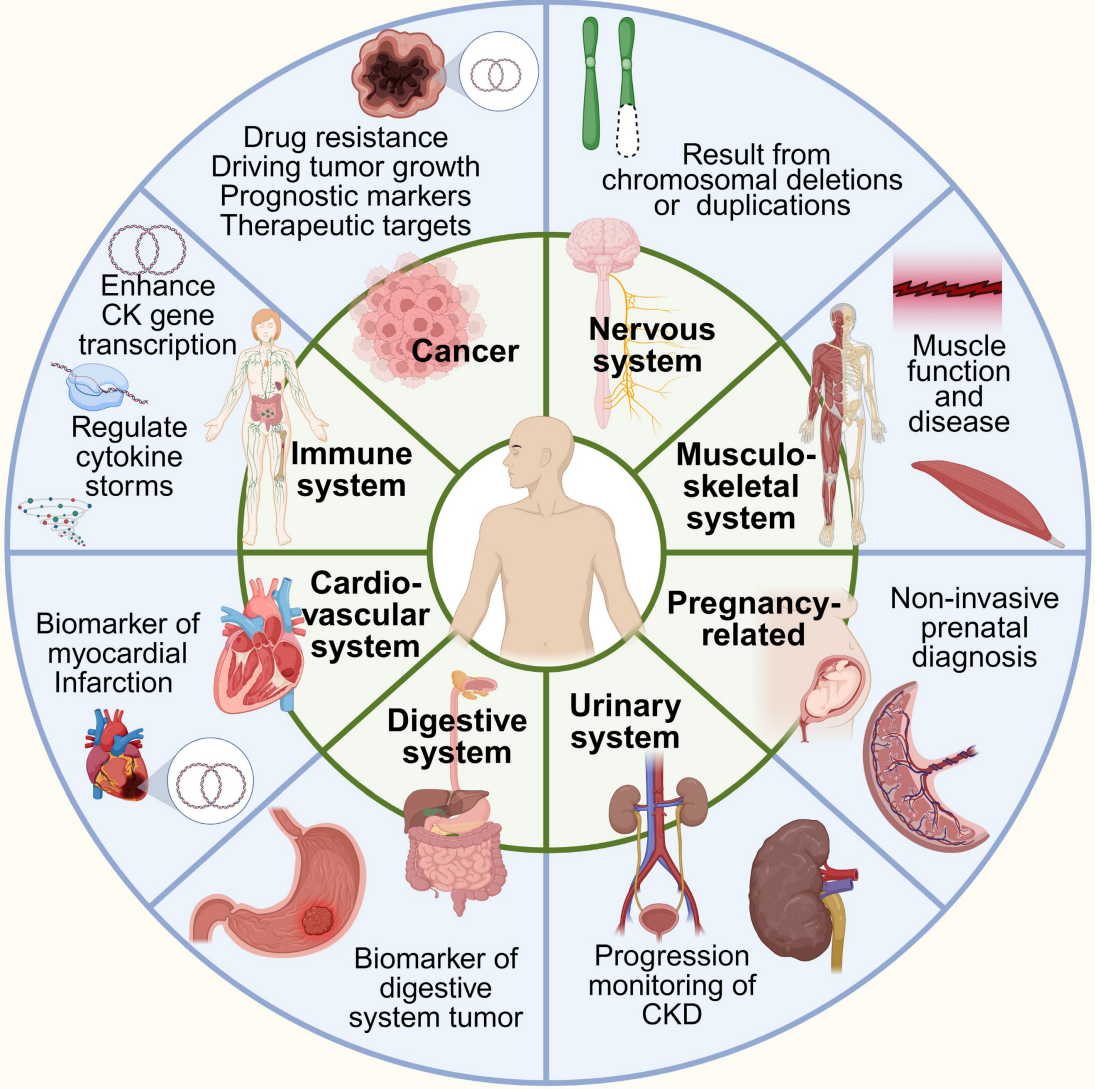
** Distribution of eccDNA in various tissues and organs of the human body.** EccDNA is associated with multiple physiological and pathological processes, including cancer progression, immune regulation, neurological diseases, muscle function, and pregnancy-related disorders. It can serve as a biomarker for conditions such as myocardial infarction and digestive system tumors. Additionally, eccDNA may result from chromosomal deletions or duplications and play a role in oncogene amplification and gene transcription regulation.

EccDNA is widely distributed in human tissues with different disease conditions, with its abundance and roles varying significantly depending on tissue type, physiological states, and pathological conditions. Recent studies reveal that eccDNA plays diverse roles, ranging from maintaining genomic plasticity in normal cells to driving oncogene amplification in tumors. Below, we examine its distribution and significance in key tissue types and disease contexts (**Figure 7**, **Table 2**).

### Elevated presence of eccDNA in cancer tissues

EccDNA is particularly enriched in cancer tissues, where it contributes to tumorigenesis by promoting oncogene amplification, enhancing tumor heterogeneity, and enabling adaptation under therapeutic pressure. High levels of eccDNA have been observed in aggressive tumors such as glioblastoma, neuroblastoma, ovarian cancer, prostate cancer, colorectal cancer, and breast cancer 27,47,128,168,169. In these cancers, eccDNA frequently harbors amplified oncogenes including, EGFR, MYC, SPOCK1, DNMT1, TP53 and GATA3, thereby supporting rapid proliferation, metabolic reprogramming, and escape from cell cycle checkpoints.

Unlike chromosomal DNA, eccDNA lacks centromeres and telomeres, allowing autonomous replication and segregation, which leads to unequal inheritance and contributes to intratumoral heterogeneity. This genomic plasticity facilitates clonal selection and the emergence of drug-resistant subpopulations under therapeutic stress—a process consistent with the "stress selection theory." For example, under targeted therapy, tumor cells that carry eccDNA enriched with resistance-associated genes (e.g., MET, ABCB1, or mutant EGFR alleles) may be preferentially selected, contributing to disease relapse and progression.

In addition to intracellular effects, eccDNA can be released into the circulation, making it a valuable biomarker for liquid biopsy. In ovarian cancer and gastric cardia adenocarcinoma, plasma eccDNA levels correlate with tumor burden and clinical progression 170,171. This suggests that eccDNA not only reflects genomic alterations within tumors but may also serve as a dynamic indicator of tumor response to treatment.

### Physiological distribution and roles of eccDNA

EccDNA is not confined to pathological contexts and has been detected in various healthy tissues such as skeletal muscle, blood, and liver 13,19,71. In normal cells, eccDNA may originate from DNA repair activities (e.g., non-homologous end joining) or chromatin remodeling events. Subtypes such as microDNA often derive from gene-rich regions, including transcription start sites and CpG islands, and may influence gene expression through epigenetic modulation or sequestration of transcription factors 17,138.

In immune cells, eccDNA plays immunomodulatory roles, especially under stress or during apoptosis. EccDNA enriched with repetitive sequences can activate the cGAS-STING signaling pathway, triggering innate immune responses 30,172-175. This immunostimulatory property positions eccDNA as both a byproduct of cellular processes and an active participant in inflammatory signaling.

### Tissue- and disease-specific roles of eccDNA

The role of eccDNA extends beyond oncology into various organ systems. In neural tissues, eccDNA has been detected in both healthy neurons and brain tumors. In pediatric gliomas, eccDNA carrying oncogenes such as EGFR and MYC is linked to tumor progression, resistance to radiation and chemotherapy, and enhanced tumor plasticity 24,47. In aging neurons, eccDNA may participate in genomic mosaicism and contribute to the accumulation of somatic variations implicated in neurodegenerative diseases 19,134,176.

In the cardiovascular system, elevated eccDNA levels have been reported following myocardial infarction (MI), with certain eccDNA fragments associated with genes involved in apoptosis (BAX, TP53) and inflammation (IL6, TNF) 177,178. The increased plasma eccDNA post-MI reflects cellular stress and necrosis, supporting its use as a prognostic marker. This enrichment may also follow a selective pattern shaped by ischemic microenvironments, where stress-selective pressure favors release of eccDNA from specific cell populations.

In the musculoskeletal system, plasma eccDNA from patients with gouty arthritis is enriched with genes associated with uric acid metabolism and inflammation, including COL1A1 and EPB42 179. These findings underscore the functional connection between eccDNA and disease-specific pathways and suggest that eccDNA may actively participate in the pathophysiology of chronic inflammatory conditions.

Beyond musculoskeletal disorders, emerging studies have identified eccDNA in other non-cancerous disease contexts. Recent evidence suggests that cf-eccDNA may serve as a disease-specific biomarker in autoimmune disorders such as systemic lupus erythematosus (SLE) 175. In patients with DNASE1L3 deficiency, cf-eccDNA exhibits distinct patterns in abundance and genomic origin, with 93 gene-derived fragments uniquely detected in affected individuals. Among these, the transcription factor BARX2 emerged as a predominant source of eccDNA. Functional associations implicate pathways related to TORC1 signaling, chondrocyte development, and immune dysregulation, including lymphopenia 175. These findings point to a potential role for cf-eccDNA in SLE pathogenesis and highlight its promise as a biomarker in autoimmune rheumatic diseases.

In metabolic disorders, such as newly diagnosed type 2 diabetes mellitus (T2DM) patients, a novel circulating eccDNA, SORBS1-circle, is markedly elevated in patients and correlates positively with key indicators of metabolic dysfunction, including HbA1c and homeostatic model assessment for insulin resistance (HOMA-IR) 126. Functional enrichment suggests a strong association between eccDNA-related genes and pathways involved in insulin resistance. Furthermore, elevated SORBS1-circle levels have been linked to hepatocyte insulin resistance under glucolipotoxic conditions, potentially driven by apoptotic DNA fragmentation. These findings highlight a previously unrecognized layer of epigenomic regulation in early-stage T2DM and position SORBS1-circle as a potential biomarker and mechanistic contributor to metabolic dysregulation.

Finally, in aging and age-related diseases, accumulation of eccDNA has been reported in senescent cells and aged tissues. Studies have shown that age-related eccDNA species often contain repetitive elements and transposable sequences, which may activate inflammatory responses or contribute to age-associated genomic instability 60,180,181. These results support the idea that eccDNA is not only a byproduct but also a modulator of aging processes.

EccDNA is ubiquitously present in human tissues and exerts diverse biological roles depending on context. In cancer, its autonomous replication and capacity to carry oncogenes underscore its pivotal role in tumor evolution, heterogeneity, and resistance. The interplay between eccDNA dynamics and tissue-specific stressors—such as hypoxia, inflammation, and therapeutic pressure—supports the application of the "stress selection theory" in understanding eccDNA enrichment in specific disease states. Moreover, its presence in normal tissues and in circulation highlights its dual identity as a functional biomolecule and a potential biomarker. As research advances, further elucidation of roles of eccDNA in different organs and under diverse pathological conditions may offer novel avenues for diagnostics and targeted therapeutics.

## Prospects and challenges

EccDNA investigations have accelerated as technological advancements uncover its essential involvement in numerous physiological and pathological mechanisms. However, significant challenges remain in fully deciphering its biological functions and potential clinical applications. In the following sections, we outline critical challenges associated with eccDNA and suggest avenues for future exploration.

### Unresolved challenges and limitations

#### Biogenesis mechanisms remain incompletely understood

Although eccDNA formation through DSBs, chromothripsis, and the BFB cycle is well-established, many mechanistic details remain unclear. Specifically, the sequence determinants that govern why certain genomic regions are preferentially excised and circularized are poorly understood. Furthermore, it remains to be elucidated whether eccDNA formation is a constitutive feature of certain cell types or induced by stressors such as DNA damage, senescence, or developmental transitions 10,94. The chromatin context, histone modifications, and potential roles of DNA-binding proteins in eccDNA biogenesis represent promising but currently underexplored avenues.

#### Technological limitations hinder comprehensive detection and analysis

Despite the availability of tools such as Circle-Seq, Circle-Map, and ECCsplorer, limitations in detection sensitivity and specificity restrict their utility, especially in non-model organisms or low-input samples 65,74,182. In tissues like the heart and brain, eccDNA abundance is often underestimated due to suboptimal detection thresholds and amplification biases. Additionally, false positives resulting from linear DNA contamination or mapping artifacts complicate interpretation. Novel approaches integrating long-read sequencing technologies (e.g., PacBio, Nanopore) and artificial intelligence models like ecSeg and Ecdetect have shown potential to improve analytical performance, reduce false positives, and handle low-coverage datasets more reliably 183-186.

#### Non-cancer-related functions remain underexplored

The role of eccDNA in cancer has been extensively documented, especially its function in amplifying oncogenes such as MYC and EGFR, which contributes to tumor heterogeneity and drug resistance 37,66,187. In contrast, its functions in physiological contexts are less well characterized. Studies in immune cells suggest that eccDNA may act as a damage-associated molecular pattern (DAMP), activating cGAS-STING signaling and triggering cytokine responses 30. In neural tissue, eccDNA has been associated with somatic mosaicism, potentially contributing to neurodevelopmental and neurodegenerative diseases 69,188. It is plausible that eccDNA could influence transcriptional plasticity in terminally differentiated cells or act as an epigenetic reservoir under stress conditions 17.

#### Clinical applications remain limited and need further validation

Although eccDNA shows potential as a disease biomarker, its clinical translation remains nascent. For example, myocardial infarction-related eccDNA (MIRECDs) identified via Circle-Seq have shown strong correlations with clinical outcomes 124, and pregnancy-specific eccDNA fragments have been explored for non-invasive prenatal testing 76,189,190. However, these findings require large-scale validation to account for biological variability and establish clinically relevant thresholds. Furthermore, therapeutic strategies targeting eccDNA, such as hydroxyurea treatment to reduce eccDNA burden in tumor cells 10,150, are promising but require rigorous evaluation in controlled trials to determine efficacy, specificity, and safety profiles.

### Future directions and opportunities

#### Enhancing detection methods and computational tools

Next-generation sequencing technologies, particularly long-read platforms and single-cell multiomics (e.g., scEC&T-seq), can provide deeper insights into eccDNA structure and tissue-specific expression profiles 10,185. There is a pressing need to improve enrichment protocols, increase detection sensitivity from low-input samples, and develop more robust bioinformatics pipelines. Integrating artificial intelligence and machine learning into analysis workflows—such as with Ecdetect or ecSeg—can significantly improve precision and consistency across datasets 68,186. Establishing benchmark datasets and automated quality control criteria will further support reproducibility and cross-study comparisons.

#### Investigating tissue-specific biological functions

Future research should prioritize the investigation of eccDNA in non-cancer contexts, particularly in the nervous, cardiovascular, and immune systems. In neural tissues, eccDNA may play a role in neuronal diversity and plasticity, potentially contributing to developmental disorders or age-related decline 69,188. In the heart, it is worth exploring whether eccDNA participates in injury responses or regenerative signaling cascades. Within the immune system, eccDNA may influence cytokine production, immune memory, or tolerance mechanisms through its interaction with pattern recognition receptors and chromatin regulators 30.

#### Expanding clinical applications in disease diagnostics and therapy

EccDNA presents a promising platform for liquid biopsy applications in cancer, cardiovascular diseases, and pregnancy-related complications. The non-invasive nature of eccDNA detection offers advantages over traditional biomarkers. For example, maternal plasma-derived eccDNA fragments have been studied as early indicators of pregnancy complications 76,189,190, while eccDNA profiles in tumors provide a means to track clonal evolution and treatment resistance 37,66,187. Therapeutic interventions that reduce eccDNA load or block its formation—such as low-dose hydroxyurea—represent novel strategies for personalized treatment 10,191. However, clinical translation will require multi-center trials, robust validation pipelines, and regulatory guidelines.

#### Establishing standardized protocols across research and clinical settings

Standardization is essential for enabling reproducible and scalable eccDNA research. This includes harmonizing sample processing protocols, such as tissue preservation, DNA extraction, and enrichment methods, as well as establishing consensus computational workflows with validated thresholds for circularity and noise reduction. The development of orthogonal validation approaches (e.g., FISH, qPCR, or CRISPR-based excision assays) is crucial for confirming eccDNA identity and function. Databases such as TeCD 63,142, when expanded and curated under international consortia, could support large-scale meta-analyses and facilitate data sharing akin to ENCODE or GTEx projects.

EccDNA represents a unique and multifaceted genetic element with important implications for genomic regulation, disease progression, and potential clinical applications. Despite advances in detection and cancer-focused research, critical challenges remain in understanding its formation, physiological roles, and translational relevance. Addressing these gaps will require collaborative, interdisciplinary efforts focused on technological innovation, functional validation, and standardized protocols. With sustained progress, eccDNA has the potential to significantly expand our understanding of genome plasticity and its role in health and disease.

## Conclusion

EccDNA has emerged as a dynamic regulator of genome plasticity, gene expression, and cellular adaptation across diverse physiological and pathological contexts. Far from being passive byproducts of genomic instability, eccDNAs serve as potent modulators of oncogene amplification, chromatin architecture, and innate immune activation. Their structural autonomy, functional versatility, and widespread distribution in both healthy and diseased tissues underscore their biological significance.

Notably, eccDNAs are increasingly recognized as promising biomarkers for disease detection and monitoring. Their presence in circulation—carrying disease-specific genomic content—offers a minimally invasive window into cellular stress responses and clonal dynamics, particularly in cancer, cardiovascular disease, and metabolic disorders. The distinct eccDNA profiles observed across tissue types suggest that eccDNA may also encode tissue-specific regulatory programs, with implications for precision diagnostics.

Collectively, current findings position eccDNA not only as an indicator of genomic stress but as a functional entity central to cellular resilience and disease progression. Continued investigation into eccDNA biology is poised to redefine our understanding of non-chromosomal genetic elements and their role in shaping phenotypic plasticity and therapeutic resistance.

## Figures and Tables

**Table 1:** Comparative features of eccDNA and related genetic molecules.

Molecule	Structure	Origin	Replication mechanism	Primary function	Disease association
eccDNA	Circular, dsDNA	Nuclear (endogenous)	Autonomous, cell-cycle-independent	Oncogene amplification, enhancer regulation	Tumor heterogeneity, drug resistance
gDNA	Linear, dsDNA	Nuclear	Chromosome replication	Genetic blueprint	Mutation-related diseases
CircRNA	Circular, ssRNA	Pre-mRNA (back-splicing)	Transcriptional output	miRNA sponge, translation modulation	Neurological disorders, cancer
Plasmid DNA	Circular, dsDNA	Prokaryotic cytoplasm	Independent	Antibiotic resistance, gene transfer	Bacterial evolution
mtDNA	Circular, dsDNA	Mitochondrial	Mitochondrial replication	Energy production	Mitochondrial diseases
Viral Circular DNA	Circular, dsDNA	Exogenous (virus)	Host machinery-dependent	Viral replication, host transformation	HPV/HBV-related cancers
ctDNA	Fragmented, dsDNA	Tumor cell apoptosis	Passive release	Liquid biopsy, tumor profiling	Cancer detection & monitoring

**Table 2:** The association of eccDNA in clinical diseases.

Disease type	Condition	Sample source	Key eccDNA feature / function	Potential application	References
Cancer	Glioblastoma	Tumor tissue	EGFRvIII ecDNA; promotes therapy resistance	Resistance monitoring	47
	Neuroblastoma	Tumor tissue	MYCN-ecDNA; drives tumor growth	Prognosis, therapy target	27
	Prostate cancer	Tumor tissue and cell	SPOCK1-eccDNA	Therapeutic target	168
	Lung and ovarian cancer	Plasma	Longer cell-free microDNA	Diagnosis	170
	Ovarian cancer	Primary and metastatic tissues	DNMT1 ^circle10302690-10302961^	Biomarker or therapeutic target	169
	Colorectal cancer	Tumor tissue	ecDNA^chr8:6495074-114379093^	Prognosis	160
	Gastric cardia adenocarcinoma	Plasma	ERBB2 focal amplifications	Prognostic marker	171
	Luminal breast cancer	Tumor tissue	TP53-ecDNA; GATA3-ecDNA	Biomarker	64
	Melanoma	Tumor tissue	LRP1B-ecDNA	Biomarker	64
	Endometrial carcinoma	Tumor tissue	TP53-ecDNA	Biomarker	64
	Bladder cancer	Tumor tissue	RB1-ecDNA	Biomarker	64
Cardiovascular	Myocardial infarction	Plasma	eccDNA accumulation	Prognosis	178
	Dilated cardiomyopathy	Cardiac tissue	Lagre and small eccDNAs	Mechanism revelation	166
Neurological	Brain aging / degeneration	Brain tissue	eccDNA accumulation; somatic mosaicism	Aging biomarker	176
Autoimmune	Systemic lupus erythematosus	Peripheral blood	eccDNA with BARX2	Rheumatic marker	175
Metabolic	Type 2 diabete	Peripheral blood	eccDNA with SORBS1	Biomarker	165
	Gestational diabetes	Plasma	PRDM16-circle	Early diagnosis	167
Musculoskeletal	Gouty arthritis	Plasma	eccDNA enriched in COL1A1, uric acid genes	Inflammatory activity marker	179
	Age-related osteoporosis	Bone tissue	eccDNA enriched in the Hippo pathway, PI3K-Akt pathway	Aging biomarker	19
Urinary system	Advanced chronic kidney disease (CKD)	Urine	eccDNA with miRNAs	Progression monitoring	124
Pregnancy-related	Fetal growth restriction	Maternal plasma	enhancer regions, immune-related pathways	Non-invasive prenatal diagnosis	190,191

## Abbreviations

DNA: Deoxyribonucleic acid
EccDNA: extrachromosomal circular DNA
DM: double minute
CsCl: cesium chloride
DHFR: dihydrofolate reductase
UTR: untranslated region
ecDNA: extrachromosomal DNA
spcDNA: small polydisperse circular DNA
ERC: extrachromosomal rDNA circle
gDNA: genomic DNA
circRNA: circular RNA
mtDNA: mitochondrial DNA
ctDNA: circulating tumor DNA
cf-eccDNA: circulating free-eccDNA
DSB: Double-strand break
NHEJ: non-homologous end joining
HR: homologous recombination
BFB: breakage-fusion-bridge
MMEJ: microhomology-mediated end joining
SSBR: single-strand break repair
LIG3: DNA ligase 3
HSR: homologous stained region
NGS: next-generation sequencing
EM: Electron microscopy
OM: Optical microscopy
3D-SIM: three-dimensional structured illumination microscopy
SEM/TEM: scanning/transmission electron microscopy
FISH: fluorescence *in situ* hybridization
LAMA: ligase-assisted mini-circle accumulation
CRISPRi: CRISPR interference
miRNA: microRNA
RISC: RNA-induced silencing complex
IFN: type I interferon
dsRNA: double-stranded RNA
siRNA: small interfering RNA
MI: myocardial infarction
SLE: systemic lupus erythematosus
T2DM: type 2 diabetes mellitus
HOMA-IR: homeostatic model assessment for insulin resistance
DAMP: damage-associated molecular patterns
MIRECD: myocardial infarction-related eccDNA
